# Progress toward Public Access to Science

**DOI:** 10.1371/journal.pbio.0060101

**Published:** 2008-04-08

**Authors:** Harold Varmus

## Abstract

PLoS Chairman of the Board Harold Varmus applauds the newly enacted NIH public access policy as a positive step toward ensuring greater access to and better use of the scientific literature.

The National Institutes of Health (NIH) is about to cross an important threshold. Starting April 7th, the authors of research reports that describe work supported by the NIH will be required to deposit accepted manuscripts into PubMed Central (PMC), the NIH's public digital library of full-text articles, with the understanding that the articles will be freely available for all to view no later than 12 months after publication.

This is a landmark event from several perspectives. Most obviously, it further accelerates the world-wide movement toward greater access to the scientific literature, markedly increasing the number of articles freely available to read online. By taking this step, the NIH will join other funding agencies—including the Wellcome Trust, the UK Research Councils, the European Research Council, and the Howard Hughes Medical Institute—all of which have recently required their investigators to deposit publications in PMC or equivalent public libraries, such as UKPMC, within six months to a year. Since NIH-supported investigators publish about 80,000 papers each year, many of them in journals that currently do not contribute their articles to PMC, the library will soon grow at about twice its already impressive rate. With an enlarged PMC, the virtues of full-text searches and ready access will be more obvious, encouraging still greater participation by authors of work not funded by the agencies that mandate deposition. As we all know, scientists want their work to be found, read, and cited.

The new NIH policy is especially gratifying to those of us who founded the Public Library of Science eight years ago with the goal of promoting greater access to and better use of the scientific literature through libraries like PMC. Still, not all articles in PMC are accessible on the same terms or timelines, and the public libraries and the laudable new policies from funding agencies still fall short of the full potential envisioned for a digital world of science. For articles in traditional, subscription-based journals, there is normally a six- to 12-month interval between publication and posting for public access. For that reason, the libraries are inherently archival—they are useful for searching relatively recent papers, but not for browsing most of the world's newly published work. Furthermore, not every important new article will have been supported by enlightened funding agencies and fall within the reach of their mandates; those may not appear in PMC at all. The libraries are also limited as archives—the new policy is not retroactive, and few of the journals that participate in PMC have contributed their older papers. This is a pity, given the potential for preserving our scientific legacy in a searchable, digital form, especially at a time when most academic libraries are placing their old paper volumes in distant warehouses. So, for various reasons, the public libraries will remain incomplete, even with respect to recent work, until all authors—and publishers—commit to ensuring access to their work. Finally, unless authors modify their copyright agreements with journals before publication—something they are urged to do—journals will continue to retain inappropriate control over the use of their articles, which is currently confined largely to reading online for most articles in PMC.

In contrast, open-access journals, like those published by PLoS or BioMed Central, make their articles immediately and freely available in PMC, eliminating any extra work by the authors and any delay before the articles are fully accessible. Furthermore, these journals permit far greater use of their articles, by allowing readers to explore and reuse the texts under the terms of a Creative Commons license. These degrees of freedom are possible because access and use do not diminish revenues: open-access publishers recover their costs upfront, frequently by charging a publication fee that is paid from research expenses, rather than with subscription charges to libraries and readers. Thus the distribution and reuse of open-access content can be without limit, just as scientists and the public would wish.

The issue of ownership of published scientific papers is a vexing one, and it could pose difficulties for another recent and exciting initiative that promises to enlarge access to scholarly work. Last month, Harvard's Faculty of Arts and Sciences (FAS) voted unanimously to require that its members provide the university with a nonexclusive license to post all their accepted articles on an openly accessible, university-maintained Web site. Because the policy might prevent some faculty, especially scientists, from publishing in journals that will not allow early free access, the policy was written to include an “opt-out” provision. This is, of course, not ideal, but much better than a policy that asks faculty to “opt-in.” Moreover, the nuisance of writing to the Provost every time a desired journal refuses to conform to the Harvard policy may cause faculty members to rethink their choice of venue, thereby minimizing use of the “opt-out” option.

As savvy journals will soon recognize, if faculty members choose to publish in other journals to comply with the new Harvard policy, the consequences will be significant—to be respected, journals need respected authors. Nevertheless, in a news article about the new Harvard policy in *Science*, former Congresswoman Patricia Schroeder, the chief lobbyist for the Association of American Publishers, says that, in view of the policy, “publishers may not be quite as excited to take articles from Harvard”[1]. This seems very unlikely, especially if the Harvard FAS is joined by other Harvard faculties and those on other prestigious campuses, where similar policies are under consideration.

The ownership issues are also not new. A decade ago, the American Academy of Arts and Sciences proposed that the nation's academic work could be made more widely available through posting on university web sites. In a subsequent Policy Forum in *Science* [2], the authors of the Academy report recognized that this could not happen without recommended reform of copyright practices. Unfortunately, little progress has been made, largely because, then as now, traditional publishers fear major losses of subscription revenues if their journals' articles are made freely available at the time of publication. Such losses are, of course, not going to occur if only some Harvard professors post their work in the university repository; but signs now point to more widespread participation in the United States, and some European institutions have already adopted such practices.

Open-access publishing offers a way out of this dilemma in academia, just as it offers solutions to the shortcomings of public libraries like PMC. When costs of publication are recovered from publishing fees instead of from subscriptions, and when authors retain copyrights and grant licenses to publishers, both of which happen with open-access publishing, then articles can be placed immediately in open university repositories (or in public libraries) without threats to revenues or infringements of ownership. We at PLoS celebrate these principles, while also applauding the new policies at Harvard, the NIH, and elsewhere, as welcome signs of continued progress toward public access to research literature.
